# An Automatic Immunoaffinity Pretreatment of Deoxynivalenol Coupled with UPLC-UV Analysis

**DOI:** 10.3390/toxins14020093

**Published:** 2022-01-25

**Authors:** Hongmei Liu, Zhihong Xuan, Jin Ye, Jinnan Chen, Meng Wang, Stephan Freitag, Rudolf Krska, Zehuan Liu, Li Li, Yu Wu, Songxue Wang

**Affiliations:** 1 Academy of National Food and Strategic Reserves Administration, No. 11 Baiwanzhuang Street, Xicheng District, Beijing 100037, China; lhm@ags.ac.cn (H.L.); xzh@ags.ac.cn (Z.X.); cjn@ags.ac.cn (J.C.); haiyangzhixin2006@163.com (Z.L.); ll@ags.ac.cn (L.L.); wyu@ags.ac.cn (Y.W.); 2 Institute of Quality Standard and Testing Technology, Beijing Academy of Agriculture and Forestry Sciences, Beijing 100097, China; wangm@brcast.org.cn; 3 Institute of Bioanalytics and Agro-Metabolomics, Department of Agrobiotechnology (IFA-Tulln), University of Natural Resources and Life Sciences Vienna (BOKU), 3430 Tulln, Austria; stephan.freitag@boku.ac.at (S.F.); rudolf.krska@boku.ac.at (R.K.); 4 Institute for Global Food Security, School of Biological Sciences, Queen’s University Belfast, Belfast BT9 5DL, Northern Ireland, UK; 5 College of Food Science and Engineering, Central South University of Forestry and Technology, Changsha 410004, China

**Keywords:** deoxynivalenol, immunoaffinity magnetic beads, automatic, UPLC-UV

## Abstract

An immunoaffinity magnetic beads (IMBs) based automatic pretreatment method was developed for the quantitative analysis of deoxynivalenol (DON) by ultra-performance liquid chromatography and ultraviolet detector (UPLC-UV). First, N-hydroxysuccinimide-terminated magnetic beads (NHS-MBs) with good magnetic responsivity and dispersibility were synthesized and characterized by optical microscopy, scanning electron microscopy (SEM), and laser diffraction-based particle size analyzer. Then, the amino groups of anti-DON monoclonal antibody (mAb) and the NHS groups of NHS-MBs were linked by covalent bonds to prepare IMB, without any activation reagent. The essential factors affecting the binding and elution of DON were meticulously tuned. Under optimal conditions, DON could be extracted from a real sample and eluted from IMB by water, enabling environmentally friendly and green analysis. Hence, there was no need for dilution or evaporation prior to UPLC-UV analysis. DON in 20 samples could be purified and concentrated within 30 min by the mycotoxin automated purification instrument (MAPI), allowing for automated, green, high-throughput and simple clean-up. Recoveries at four distinct spiking levels in corn and wheat ranged from 92.0% to 109.5% with good relative standard deviations (RSD, 2.1–7.0%). Comparing the test results of IAC and IMB in commercial samples demonstrated the reliability and superiority of IMB for quantitatively analyzing massive samples.

## 1. Introduction

The primary representative of the type B trichothecenes, deoxynivalenol (DON), commonly called vomitoxin, is generated by some *Fusarium* species [1]. DON is one of the most prevalent mycotoxins in various cereals crops and processed grains, such as corn, wheat, bread, beer, and malt [2,3,4,5], with a detection rate of 57% in 11,444 tested samples [6]. The consumption of grains or food products contaminated with DON could result in various adverse effects to humans and animals, including feed refusal, gastroenteritis, nutrient malabsorption, diarrhea, vomiting, teratogenicity, immunotoxicity and cardiotoxicity [7,8,9]. The International Agency for Research on Cancer has categorized DON in the group 3 as not classifiable regarding their carcinogenicity to humans since 1993 [8]. Thus, many countries have established the maximum level (ML) for DON in cereals and its derived products. The ML of DON in Commission Regulation (EC) No 1881/2006 ranged from 200 μg/kg (processed cereal-based foods and baby foods for infants and young children) to 1750 μg/kg (unprocessed durum wheat, oats and maize) [10]. The ML of DON in China was 1000 μg/kg in cereals and related products [11]. In addition, the Joint FAO/WHO Expert Committee on Food Additives recommended a daily dose of 1 mg/kg body weight. Furthermore, a tolerable maximum daily dose of 1 mg DON per kilogram of body weight was recommended by the Joint FAO/WHO Expert Committee on Food Additives [12].

DON, a polar organic compound, is stable in processing and cooking, thus to avoid the potential health risks it is important to evaluate its contamination levels [13]. High-performance liquid chromatography (HPLC) with ultraviolet (UV) [3,13,14] or mass spectrometry (MS) [7,15] and gas chromatography with electron capture [16] or MS detection [17] were the most prevalent approaches for the quantitative assessment of DON in food and feed. To eliminate interfering matrix chemicals, concentrate DON and protect the analytical instrument, various clean-up approaches have been utilized after the solid–liquid extraction of DON, such as dispersive solid-phase extraction (DSPE) [15] immunoaffinity columns (IACs) [2,3,5,13,14] or multifunctional clean-up columns [18]. Among them, the IAC, on the basis of the selective interaction between DON and its antibodies, is one of the most popular and effective sample pretreatment methods. However, using IAC as a pretreatment method is less well-adapted to high-throughput screening of DON because it necessitates tedious and time-consuming operation procedures, expensive use costs, and highly skilled technicians.

With the increasing desire to improve pretreatment efficiency and reduce testing costs, major efforts have been dedicated to high-throughput and automatic sample pretreatment methods. Immunoaffinity magnetic beads (IMBs), with magnetic beads (MBs) as carriers of specific antibodies, provide an alternative immunoaffinity separation technique to conventional IACs, due to the high surface-to-volume ratio and superparamagnetism of the IMBs [19]. Compared with IACs, IMBs have excellent dispersibility in a liquid medium, which increases the opportunities for the interaction with their targets. Hence, IMBs can be used to simply, rapidly and specifically capture and separate targets from complex sample matrices through an exterior magnetic field, with no need for centrifugation or filtration [20]. The applicability of IMB has been widely demonstrated in diagnostic techniques [21] and protein purification [22]. Furthermore, an automated and high-throughput sample pretreatment method for ochratoxin A and aflatoxins was built on the basis of their corresponding IMBs in our previous works [23,24]. Although the detection level and rate of DON were much higher than those of ochratoxin A and aflatoxins [4,6], the pretreatment of DON has yet to be automated for high throughput.

In the present study, an automated and high-throughput pretreatment method was developed for highly efficient and selective isolation and concentration of DON on the basis of anti-DON IMB. N-hydroxysuccinimide-terminated magnetic beads (NHS-MBs) with good magnetic responsivity and dispersibility were synthesized and used as carriers of the anti-DON monoclonal antibody (mAb), rather than the conventional agarose in IACs and the traditional MB with carboxyl or amino groups. The factors affecting the incubation with the IMBs and elution of DON from the IMBs were meticulously tuned for efficient and selective isolation and concentration of DON. The mycotoxin automatic purification instrument (MAPI) was used to achieve high-throughput, easy and automatic sample pretreatment, with its 20 pairs of magnetic sticks and plastic coats. Water was used to extract DON from naturally contaminated samples [2,3], as well as to elute DON from IMBs instead of methanol and acetonitrile, enabling environmental safe and green analysis. Hence, there is no need for dilution or evaporation of the eluent prior to UPLC-UV analysis, thereby further improving sample handling efficiency. In the end, the applicability of IMBs for simple, green, automated and high-throughput clean-up and the subsequent determination of DON by UPLC-UV was demonstrated by analyzing naturally contaminated samples and DON reference materials.

## 2. Results and Discussion

### 2.1. Characterization of the MBs and IMBs

**Figure 1:**
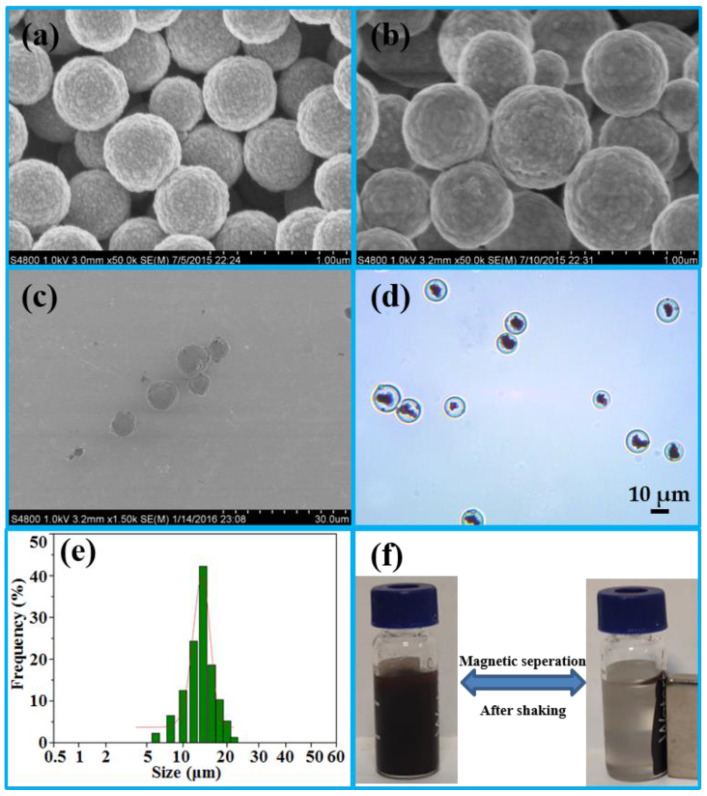
SEM images of Fe_3_O_4_ (**a**), Fe_3_O_4_·SiO_2_ (**b**) and IMB (**c**); optical microscopy picture of the IMB (**d**); size distribution graph of the IMB (**e**); paramagnetism of the IMB (**f**). (SEM: scanning electron microscopy; IMB: immunoaffinity magnetic beads).

SEM was first used to characterize the geometry and morphology of Fe_3_O_4_, Fe_3_O_4_·SiO_2_, and IMB. Compared with Figure 1a (Fe_3_O_4_), the surface of Fe_3_O_4_ was coated with a smooth layer of silica, as shown in Figure 1b, which could improve hydrophilicity, dispersion and stability [20]. The SEM image in Figure 1c of the IMB exhibited excellent monodispersity. The core of the IMB was the clusters of Fe_3_O_4_·SiO_2_ in Figure 1d, which were uniformly entrapped within the agarose hydrogel either in an aggregated or dispersed form to further enhance the hydrophilicity and stability of the IMB. The size of the IMB in Figure 1e was in the range of 5 μm to 25 μm, most of which was approximately 15 μm, detected a by laser diffraction-based particle size analyzer. Because of the clusters of Fe_3_O_4_·SiO_2_, the scattered IMBs in an external magnetic field could accumulate within 20 s (Figure 1f), before dispersing quickly with minor shaking once the magnetic field was removed, confirming its superior magnetic responsivity and dispersibility.

The content of NHS on the surface of NHS-MB was quantified based on a spectrophotometric assay, according to a previous report [25]. The principle for the assay was that N-hydroxysuccinimide was ionized under 0.1 M NH_4_OH basic conditions, whose absorption spectrum at 260 nm was increased. Firstly, a series of NHS standard solutions (0.2 mM, 0.5 mM, 1.0 mM, 1.2 mM, 1.4 mM, 1.8 mM, 2.0 mM) was configured by 0.1 M NH_4_OH, whose absorbance was measured in triplicate at 260 nm. The concentration (*x*) of NHS and absorbance at 260 nm (*y*) were used to draw the standard curve (*y* = 0.6583*x* + 0.051, with R^2^ = 0.9981). Secondly, to prevent the rapid hydrolysis of NHS, 1 mL of NHS-MB (10%, *v*/*v*) was quickly washed twice with water with the aid of a magnetic stand. Then, the NHS-MB was mixed vertically and incubated with 1 mL of NH_4_OH (0.1 M) for 5 min at room temperature. After magnetic separation, the supernatant was transferred into another centrifuge tube. The supernatant was then withdrawn into the aforementioned centrifuge tube, after another 1 mL of NH_4_OH (0.1 M) was added and mixed vertically with MB for 5 min. After diluting the supernatant to 5 mL with NH_4_OH (0.1 M), the average absorbance was 1.101 at 260 nm in triplicate. Hence, 8 μmol of NHS was on the surface of 1 mL of NHS-terminated MB (10%, *v*/*v*), calculated by the standard curve of NHS (NHS(μmol) = (1.101 − 0.048)/0.6583 × 5 = 8 μmol). 

### 2.2. Synthesis of the IMB

#### 2.2.1. Screening of the mAb

**Figure 2:**
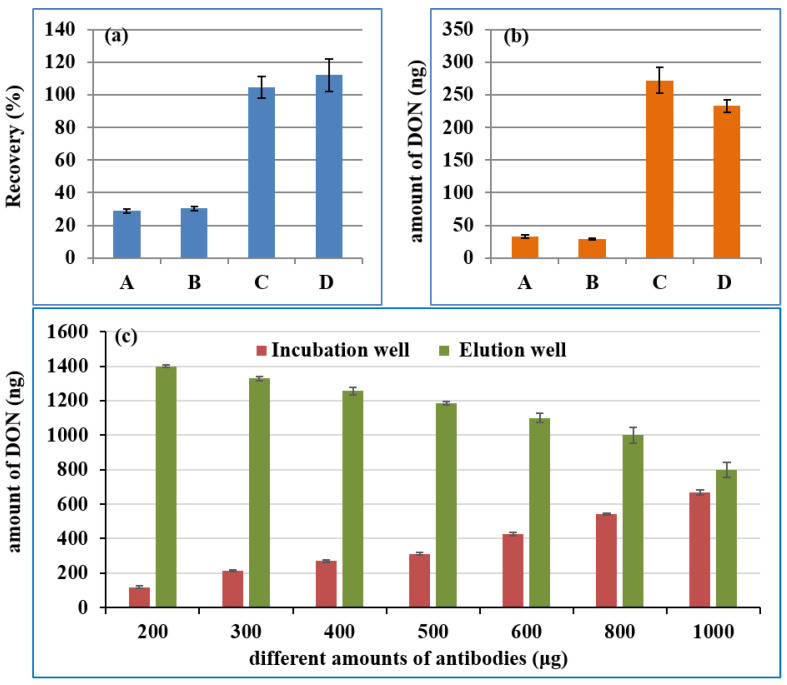
The recovery of 100 ng DON (**a**), the maximum adsorption capacity (**b**) of IMBs from different manufacturers, and the maximum adsorption capacity (**c**) of the IMB by coupling different amounts of antibodies with 0.5 mL MB. (A: Wuhan Yangda Biological Technology Co., Ltd.; B: Shandong Landu Biotechnology Co., Ltd.; C: Wuxi Chuangpu Biological Technology Co., Ltd. and D: Beijing Huaanmaike Biotechnology Co., Ltd.; DON: deoxynivalenol; IMB: immunoaffinity magnetic bead; MB: magnetic bead).

The high affinity of the mAb was another core for the IMB, which was the identification component of DON. As a carrier for mAb, NHS-MB can couple with anti-DON mAb at room temperature through the covalent bond between NHS groups on the MB and amino groups on the mAb, without the activation of EDC or glutaraldehyde. Firstly, antibodies from different manufacturers were screened by comparing their recovery rate and the maximum adsorption capacity. Different IMBs were prepared on the basis of 400 μg mAb from four manufacturers (A: Wuhan Yangda Biological Technology Co., Ltd., Wuhan, China; B: Shandong Landu Biotechnology Co., Ltd., Shandong, China; C: Wuxi Chuangpu Biological Technology Co., Ltd., Wuxi, China and D: Beijing Huaanmaike Biotechnology Co., Ltd., Beijing, China) and 2 mg NHS-terminated MB, according to the procedure in “4.5 Preparation of Anti-DON Immunoaffinity Magnetic Beads”. Then, 100 ng of DON was added to the first well to investigate the recovery rate of the IMB, while 500 ng of DON was used to assess its maximum adsorption capacity. The recovery rates for the IMB from the mAbs of C and D were close to 100%, whereas those for A and B were lower than 40%, as shown in Figure 2a. The maximum adsorption capacity in Figure 2b was C > D > A > B. Hence, the mAb from C was selected in the subsequent experiments.

#### 2.2.2. Optimization of the Coupling Conditions

The IMB was transferred between the different wells of the kit with the aid of magnetic bars. Hence, too many MBs may affect its transfer efficiency due to the limitations of well size and magnetic bars, and an insufficient amount of MBs could lead to a low mAb load. Because of the high ML (1000 μg/kg) of the DON, the low mAb load could result in the maximum adsorption capacity of IMB not meeting the requirements of actual sample detection. Therefore, to circumvent the limitations of MB concentration and mAB load, a defined amount of MBs (5 mg) was used for coupling different amounts of antibodies (200, 300, 400, 500, 600, 800 and 1000 μg). Then, 1500 ng of DON was applied to investigate the maximum adsorption capacity of 2 mg IMB. The DON in the incubation well and in the eluting well of the kit was analyzed by UPLC-UV after treatment with the MAPI. The detected amount of the DON was shown in Figure 2c. With the increase in the amount of mAb, less DON was left in the incubation well (the first well), while more DON was eluted in the elution well (the seventh well). Increasing the amount of antibody could further increase the maximum adsorption capacity of IMB, which could lead to the wasting of mAb. The maximum adsorption capacity was 670 ng, which was when 1000 μg of mAb and 0.5 mL MB were used to prepare the IMB. When 1 mL of the supernatant in “4.6. Sample sources and preparation” was added in first well of the kit, the maximum amount of DON in the real sample that could be accurately quantified was 2680 μg/kg, which was much higher than its MLs (1000 μg/kg). 

### 2.3. Optimization of the Elution Conditions

**Figure 3:**
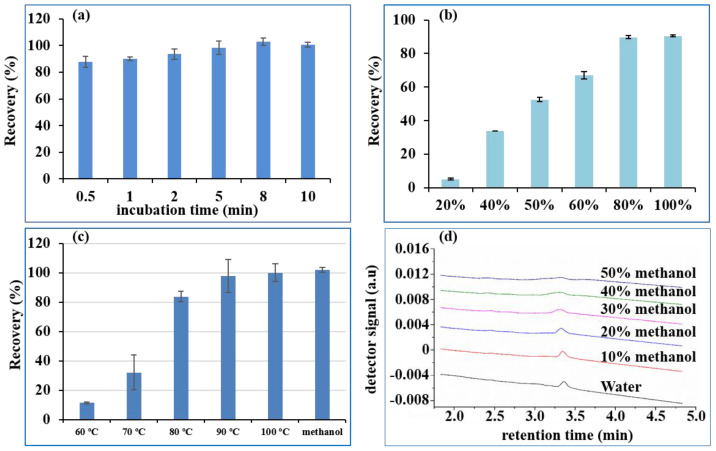
Recovery of 100 ng DON under different incubation times (**a**), methanol concentration in eluent (**b**), water at different temperatures in elution (**c**) and chromatogram of 100 ng/mL DON in different concentrations of methanol (**d**). (DON: deoxynivalenol).

Adequate time was necessary for the IMB to fully capture the DON. Hence, 100 ng of DON was added to the first well and then incubated for 0.5, 1, 2, 5, 8, and 10 min with 2 mg IMBs. The recovery in the elution well is shown in Figure 3a. The recovery of DON improved as incubation time increased within 5 min, and fluctuated around 100% from 5 min to 10 min. Hence, it only takes 5 min for the IMB to completely capture the DON, which was used as the incubation time in the subsequent experiments.

### 2.4. Optimization of the Elution Conditions

#### 2.4.1. Elution Ability of Different Concentrations of Methanol

The most commonly employed eluent for the IAC of mycotoxins is methanol [2,5,13,14], which can deactivate mAbs and desorb the target analytes from IAC. Therefore, we firstly investigated the elution ability of different concentrations of methanol in water. Different ratios of methanol-water (20 + 80 to 80 + 20) were therefore used as the eluent solvent for 2 mg IMBs loaded with 100 ng of DON, to optimize the lowest concentration of methanol in the eluent solvent. As seen in Figure 3b, increasing the methanol concentration from 20% to 60% in the elution solvent resulted in an increase in the recovery of DON from 15.0% to 76.9%, while 80% and 100% methanol in water gave recoveries of 99.7% and 100.4%, respectively. Therefore, a higher methanol content (>80%) could effectively desorb DON from the IMB, which was consistent with a reported study on IAC [26]. However, the purified DON in the eluent could not be directly injected onto the UPLC-UV system prior to evaporation and redissolution with the mobile phase, because the high quantity of methanol (>30%) rendered the peak broad, as shown in Figure 3d. However, when the methanol concentration was lower than 100%, the speed of drying by nitrogen blowing was obviously slower than that for 100% methanol, due to the presence of water. Therefore, 100% methanol was more suitable as the eluent.

#### 2.4.2. Elution Ability of Hot Water

Currently, the eluents used for IAC in application notes from manufacturers are organic solvents (either methanol or acetonitrile), which are usually not injected directly into the reversed-phase chromatographic system. The eluent was further diluted or evaporated and redissolved in the mobile phase to avoid peak broadening [5,13]. Hence, the idea to investigate an organic solvent-free eluent was initially triggered by using hot water to disrupt the affinity of the antibodies [3,27]. The cooled hot water eluent could be directly injected onto a reversed-phase liquid chromatography column without evaporation, reconstitution or dilution. Hence, the aim of this study was to automatically implement hot water as the eluent of IMB for DON, replacing organic solvents in the standard elution protocol. Elution temperature is a critical parameter to ensure quantitative elution of DON from IMB. When the IMB was transferred to the eluting well, it was automatically heated to the set temperature and kept there for 5 min. Finally, the eluent was further analyzed by UPLC. Recoveries of DON at different temperatures (Figure 3c) revealed that elution at 70 °C was ineffective (less than 20%), elution at 80 °C was still incomplete (in the range of 80% to 90%), and DON was quantitatively eluted at 90 °C. Hence, DON could be extracted and cleaned up by pure water, with no need for any organic solvent prior to chromatographic determination.

### 2.5. The Selectivity and Specificity of the IMB

**Figure 4:**
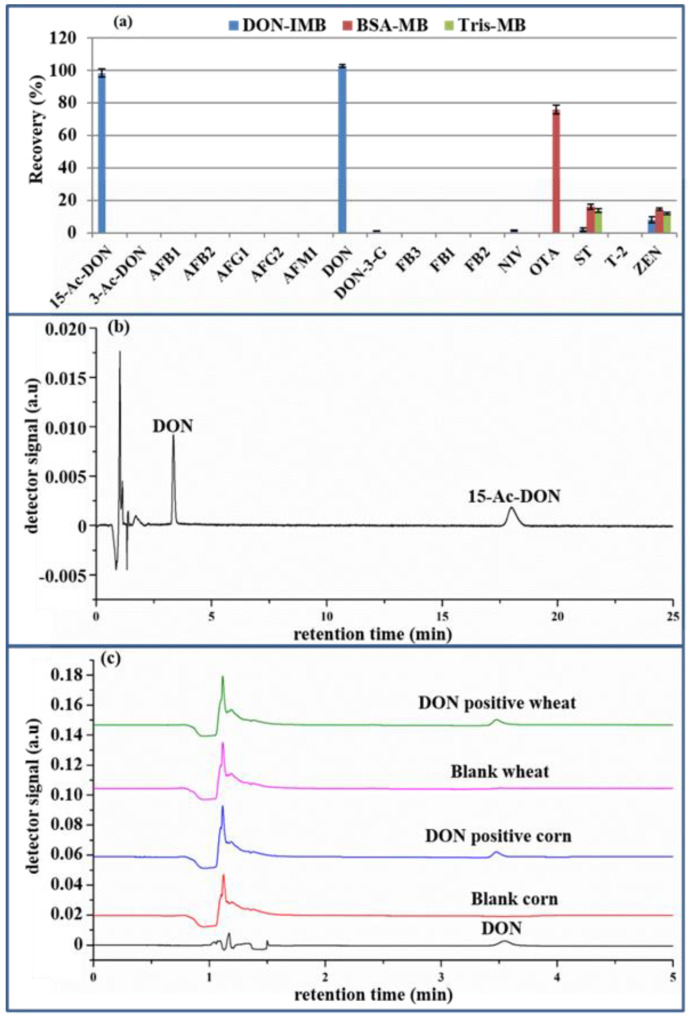
The recovery of the 17 kinds of mycotoxins pretreated with BSA-MB, Tris-MB and anti-DON IMB (**a**), the chromatogram of DON and 15-ADON (**b**) and the chromatogram of the DON standard, blank wheat and corn extracts and the positive wheat and corn extracts (**c**). (DON: deoxynivalenol; 15-ADON: 15-acetyl-deoxynivalenol BSA-MB: bovine serum albumin-magnetic bead; Tris-MB: Tris-magnetic bead; IMB: immunoaffinity magnetic bead).

The selectivity and specificity of the IMB toward DON was assessed by comparing the recoveries of DON, popular mycotoxins (i.e., aflatoxin B_1_ (AFB_1_), aflatoxin M_1_ (AFM_1_), aflatoxin B_2_ (AFB_2_), aflatoxin G_1_ (AFG_1_), aflatoxin G_2_ (AFG_2_), deoxynivalenol-3-glucoside (DON-3G), fumonisin B_1_ (FB_1_), fumonisin B_2_ (FB_2_), fumonisin B_3_ (FB_3_), nivalenol (NIV), ochratoxin A (OTA), sterigmatocystin (ST), T-2 toxin (T-2), zearalenone (ZEN)) and the structural analogs of DON (3-acetyl-deoxynivalenol (3-ADON), 15-acetyl-deoxynivalenol (15-ADON) and deoxynivalenol-3-glucoside (DON-3G)). Following the procedure outlined in “4.5. Preparation of anti-DON immunoaffinity magnetic beads,” the anti-DON mAb was substituted with BSA (0.2% BSA, 0.1 M MES, 0.15 M NaCl, pH 6.0) and tris (50 mM Tris-HCl, 0.15 M NaCl, pH 7.2) to produce BSA-MB and Tris-MB. The nonspecific test of MB was, respectively, conducted with BSA-MB and Tris-MB for the recoveries of the above 17 kinds of mycotoxins. As illustrated in Figure 4a by UHPLC-HRMS, the recoveries of ST and ZEN were in the range of 10–20% for both BSA-MB and Tris-MB and 70–80% of OTA for BSA-MB, while they were lower than 10% for the other mycotoxins. Through comparison, the 70% recovery of OTA was due to the affinity of BSA to OTA [28], while the Tris-MB had little nonspecific adsorption for common mycotoxins. Hence, 50 mM Tris-HCl, 0.15 M NaCl, pH 7.2 was utilized to block the remaining active sites of MB. Secondly, to further demonstrate the selective detection of DON, the above 17 kinds of mycotoxins, except DON, were chosen as the negative controls for the anti-DON IMB. The eluent in the 7-well was evaluated by UHPLC-HRMS. As shown in Figure 4a, the recoveries of DON and 15-ADON were approximately 100%, while those of the other toxins were lower than 10%, which were in accordance with a previous report [1]. When the DON immunogen was synthesized by coupling the carrier protein (BSA) via a linker on C3 and/or C15 of DON before immunization, the generated monoclonal antibody could recognize DON and 15-ADON, but not 3-ADON. Fortunately, this does not interfere with the quantification of DON, owing to their different retention times (3.350 min for DON and 17.997 min for 15- ADON in Figure 4b). Finally, the comparison of the chromatograms of the DON standard, blank wheat and corn extracts and the eluting solution of the positive wheat and corn extracts in Figure 4a shows no interference peak at the retention time of DON, which was clearly separated with good peak shapes. Consequently, anti-DON IMB could effectively remove matrix interference in real samples, with acceptable selectivity and specificity.

### 2.6. Analytical Performance

The performance of the developed method was thoroughly verified according to EU Regulation (No. 401/2006) [29], including linearity, LOD, LOQ, accuracy and precision. The peak area of various concentrations of DON detected by UPLC-UV obviously rose as its concentrations increased. A six-point calibration curve (*y* = 60.091*x* + 650.92, R^2^ = 0.9999) was generated between the peak area (*y*) and DON concentration (*x*) in the range of 25–1500 ng/mL. The LODs (three times of the background chromatographic noise) and LOQs (10 times of the background chromatographic noise) [23] were 40 μg/kg and 100 μg/kg, respectively, which demonstrated the excellent sensitivity of this method. The recovery rate was used to characterize the accuracy of the analytical results, while the relative standard deviation (RSD) was used to characterize the method’s precision. The detected amount and recoveries (*n* = 3) in corn and wheat were shown in Table 1 at four distinct spiking levels. Recoveries ranged from 92.0% to 109.5%, while the RSD was 2.1% to 7.0%, indicating good accuracy and precision. Hence, the aforesaid method for validation of data met all of the requirements of EU Regulation (No. 401/2006) [29], which means that the current IMB-based automatic pretreatment coupled with the UPLC-UV method was selective, precise and accurate.

### 2.7. Application in Real Samples

**Figure 5:**
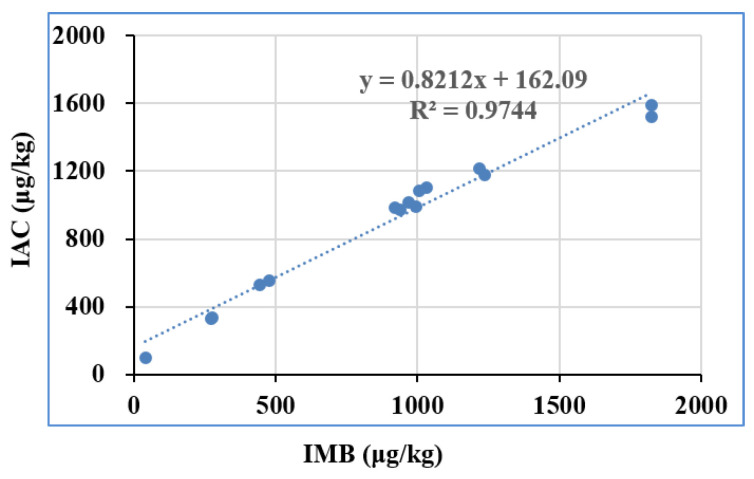
Comparison of the IMB method with the standard IAC method based on tests on real samples (IMB: immunoaffinity magnetic bead).

With the aim of validating the feasibility and applicability, 30 batches of real samples collected from local stores and markets were determined by the suggested approach under optimum settings. The extracted solution of these real samples was pretreated with IMB and IAC, and the eluent was quantified by UPLC-UV. A positive correlation (*y* = 0.8212*x* + 162.09, R^2^ = 0.9744) of the detected amount above LOQ from IMB and IAC was obtained in Figure 5, which means that the results from IMB agree well with those from the IAC method. Furthermore, the feasibility and applicability of the IMB was verified with CHARM DON reference materials, including MRM-DON-CORN-0.5-0021, MRM-DON-CORN-1-002G, MRM-DON-WHEAT-0.5-005A, and MRM-DON-WHEAT-1-010. The detected DON by UPLC-UV is shown in Table 2, which also agrees well with the assigned value, in accordance with the National Measurement Standards of China (JJF 1507–2015) [30] and the ISO Guide 33:2015 [31]. The above results indicated that the proposed method was very suitable for the determination of DON in real samples. These results further confirm that IMB could be used as a new preprocessing platform for rapid purification and concentration assays of DON.

## 3. Conclusions

An automated pretreatment method based on anti-DON IMB coupled with UPLC-UV analysis was successfully developed and implemented for the quantification of DON in naturally contaminated samples obtained from local markets in China as well as in certified reference material. NHS-MBs with excellent magnetic responsivity and dispersibility were synthesized and characterized, serving as carriers of anti-DON mAb. The antibodies were covalently bound to prepare IMB without the need for any activation reagent. Water, rather than the usual methanol or acetonitrile, was utilized to extract DON from real samples, as well as to elute DON from IMB, after meticulously tuning the binding and elution conditions. Hence, the proposed method is not only green and environmentally safe, but also eliminates the need for dilution or evaporation prior to UPLC-UV analysis. DON in 20 corn and wheat samples were analyzed in the range of 100 μg/kg to 2680 μg/kg by MAPI. The overall procedure for automatic handling of 20 samples took 30 mins demonstrating the simple and high throughput character of the proposed clean-up procedure. Under the optimal conditions, the developed method has good linearity, LODs, LOQs, accuracy, precision, and selectivity. Comparing results obtained with classic IAC clean-up and the developed IMB when analyzing commercial samples demonstrated the reliability and usability of IMB in high-throughput applications. Furthermore, this automatic pretreatment method provides a reference for other mycotoxins, environmental pollutants and pesticide residues.

## 4. Materials and Methods

### 4.1. Chemicals and Regents

Sigma-Aldrich (St. Louis, MI, USA) provided N-Hydroxysuccinimide and N-(3-dimethylamnopropyl)-N-ethylcarbodiimide hydrochloride (EDC). Morpholinoethanesulfonic acid (MES) monohydrate was provided by shanghai macklin Biochemical Technology Co., Ltd. (Shanghai, China), while bovine serum albumin (BSA) was by beijing biotopped Technology Co., Ltd. (Beijing, China). The HPLC grade of methanol (MeOH) and acetonitrile (ACN) were purchased from Fisher Scientific (Waltham, MA, USA). The Milli-Q purification system (Bedford, MA, USA) provided ultrapure water. The purified anti-DON monoclonal antibody (mAb) at 3.15 mg/mL was prepared by Wuxi Chuangpu Biological Technology Co., Ltd. (Jiangsu, China) in phosphate-buffered saline (PBS). The stock standard solution of DON and other mycotoxin standards in amber glass vials at −20 °C were bought from Romer Labs, Inc. (Union, MO, US), including 3-acetyl-deoxynivalenol (3-ADON), 15-acetyl-deoxynivalenol (15-ADON), aflatoxin B_1_ (AFB_1_), aflatoxin M_1_ (AFM_1_), aflatoxin B_2_ (AFB_2_), aflatoxin G_1_ (AFG_1_), aflatoxin G_2_ (AFG_2_), deoxynivalenol-3-glucoside (DON-3G), fumonisin B_1_ (FB_1_), fumonisin B_2_ (FB_2_), fumonisin B_3_ (FB_3_), nivalenol (NIV), ochratoxin A (OTA), sterigmatocystin (ST), T-2 toxin (T-2), zearalenone (ZEN). The above stock solution was diluted with 50% methanol/water solution to prepare the corresponding working standard solutions, which were kept in amber glass vials at 2–8 °C for a month and then renewed. The DON reference material (lot number: MRM-DON-CORN-0.5-0021, MRM-DON-CORN-1-002G, MRM-DON-WHEAT-0.5-005A, MRM-DON-WHEAT-1-010) was provided by Charm Sciences, Inc. (MA, USA). The 0.2-μm PTFE acrodisc Syringe Filters were provided by PALL (Port Washington, NY, USA). Ready-to-use PBS powder (10 mM, pH 7.4) was purchased from Boster Biological Technology Co. LTD (Wuhan, China). All additional analytical-grade chemical reagents, such as tetraethoxysilane, polyethylene glycol 8000 (PEG 8000), epichlorohydrin, iron chloride hexahydrate (FeCl_3_·6H_2_O), glycine, and agarose were provided by Shanghai Aladdin Biochemical Technology Co., LTD (Shanghai, China). To minimize DON pollution in the environment, all DON-contaminated laboratory glassware, containers and waste were soaked in 5% sodium hypochlorite for more than 24 h, prior to further washing with detergent and water, or discarding.

### 4.2. Instrument and Analytical Conditions

MTV-100 multitube vortexer (Hangzhou Aisheng instrument Co., Ltd., Hangzhou, China) was used to extract DON from samples at 2500 rpm for 20 min. Mettler toledo International Trading Co., LTD (Shanghai, China) provided the FE28 pH meter, and Sigma Laborzentrifugen GmbH (Osterode am Harz, Germany) provided the refrigerated centrifuge. The absorbance at 260 nm was measured by an ultraviolet–visible spectrophotometer (SMA4000, Merinton Instrument Inc., Beijing, China). The topology, size, and optical morphology of the MB and IMB were, respectively, studied by both an SU-70 scanning electron microscope (SEM) (Tokyo, Japan) and 6XB upright metallurgical microscopy (Shanghai optical instrument manufacture, China). The laser diffraction method (Mastersizer 3000, Malvern Inc., Malvern, UK) was used to record the particle size and distribution of the IMB. 

The 10.0 μL of samples and DON standards were analyzed by Waters ACQUITY UPLC H-class system, equipped with a binary pump, an inline degasser vacuum degasser and a UV detector at 218 nm, which was controlled by Waters EMPOWER 3 software (Waters, Manchester, UK). Chromatographical separation at 40 °C was conducted with a BEH reverse-phase C18 column (2.1 mm × 100 mm, 1.7 μm) (Waters, Manchester, UK). Iisocratic elution was carried out with acetonitrile–water (10:90, *v*/*v*) as the mobile phase in 0.3 mL/min.

Ultra-high-performance liquid chromatography coupled with high-resolution mass spectrometry (UHPLC-HRMS) was utilized to investigate the specificity of anti-DON IMB, including an UHPLC system (Ultimate 3000, Thermo Fisher Scientific, Waltham, MA, USA) and an Orbitrap mass spectrometer (Exactive™; Thermo Fisher Scientific, Waltham, MA, USA). The temperature of a CORTECS UHPLC C18 column (100 mm × 2.1 mm, 1.6 μm) (Waters, Milford, MA, USA) was set at 40 °C, with an injection volume of 2 μL. The mobile phase was set at a flow rate of 0.3 mL/min, including 0.1% formic acid and 1 mM ammonium acetate (A), and MeOH (B). The following was the gradient mode: 0–2 min: 10% B; 3 min: 20% B; 4 min: 21% B; 5–7 min: 26% B; 10.5–13.5 min: 60% B; 14.5–17 min: 95% B; 18–21 min: 10% B. MS spectra equipped with an electrospray ion (ESI) source in positive ionization mode was acquired in the full mass scan mode in the mass range of 100–800 m/z, with the resolving power set at full-width at half maximum of 70,000. The following are the detailed operational parameters: 300 °C and 320 °C for source temperature and capillary temperature, 35 and 10 arbitrary units for sheath gas and arbitrary units, and 3.2 kV for capillary voltage. The precise m/z values for the extracted ions of the 17 mycotoxins in the UHPLC–HRMS are listed in Table S1, as described in our previous reports [32]. 

### 4.3. Synthesis of Magnetic Beads

The magnetic beads centered on Fe_3_O_4_·SiO_2_ and wrapped in hydrophilic agarose were synthesized as described in our previous study [20], as shown in the Electronic Supporting Material (ESM).

### 4.4. Synthesis of N-Hydroxysuccinimide-Terminated Magnetic Beads

The MB was first modified with epoxy groups and then further activated by carboxyl groups with the aid of glycine, according to our previous method [20], which was also shown in the ESM. Finally, the carboxyl-activated MB was further functionalized with an NHS group according to a previously published approach, with a few tweaks [33,34]. 

Next, 1 mL of the carboxylated MBs was, firstly, washed extensively in anhydrous DMSO 3 times to achieve anhydrous conditions. The carboxylic group on the surface of MBs was then activated at 45 °C for 3 h with 200 mg EDC and 200 mg NHS. After incubation, the NHS-terminated MB was washed 3 times with anhydrous DMSO solution and stored in anhydrous N-N-dimethyl acetamide (DMAC) to prevent hydrolysis of the NHS group.

### 4.5. Preparation of Anti-DON Immunoaffinity Magnetic Beads 

The anti-DON mAb was coupled with NHS-terminated MB with some modifications, according to previously published methods [35]. With the use of an external magnetic field, 20 mL of NHS-terminated MB (10%, *v*/*v*) was mixed uniformly and rinsed twice with absolute ethanol. Then, 40 mg of anti-DON mAb (3.15 mg/mL, in PBS) was diluted with coupling buffer (0.1 M MES and 0.15 M NaCl in pH 6.0) to 40 mL, and then rotated at 20 rpm with NHS-MB for 2 h at room temperature. Afterward, 40 mL of blocking buffer (0.05 M Tris-HCl and 0.15 M NaCl in pH 7.2) was utilized to block the remaining NHS groups of the MB under the same conditions. Finally, the anti-DON IMB was washed twice with 40 mL washing buffer (PBS with 0.1% Tween 20 in pH 7.2), 40 mL PBS three times, and then resuspended in 20 mL PBS with 0.02% of sodium azide at 4 °C for further use.

### 4.6. Sample Sources and Preparation 

Agricultural products, including wheat and corn, were bought in local stores and supermarkets, the representative samples of which were finely milled and thoroughly homogenized in accordance with the general guidelines from the FAO and WHO about sampling [36]. Samples were extracted according to national standard GB 5009.111-2016, with some modifications [37]. Next, 5 g of representative sample, 1 g of PEG 8000 and 20 mL of ultrapure water were thoroughly vortexed at 2500 rpm for 20 min by a multitube vortexer in a centrifuge tube of 50 mL, which was subsequently centrifuged at 8000 rpm for 5 min before the clean-up operation.

### 4.7. IMB Purification

The DON purification kit was developed on the basis of anti-DON IMB with a specially tailored 7-tube strip as the reaction vessel. The MAPI with an intelligent mechanical unit was used to automatically perform the whole DON purification process. Under the coordinated control of an embedded real-time operating system, it can not only simultaneously purify and concentrate DON from 1 to 20 samples in parallel with the DON purification kit, but also meet the requirement for rapid sample processing with a lightweight design. The corresponding magnetic bar on the instrument is a quick, simple and efficient way to aggregate the IMB to the outer wall of the magnetic bar sleeve from the reaction liquid [38]. The purification and concentration of DON can be achieved in only 3 steps, by immobilizing the liquid and transferring the magnetic beads, with no need for professional technicians. Firstly, the supernatant (1 mL) was added into the first well of the kit. Secondly, the kit was put into the mycotoxin automatic purification instrument, the purification program for DON was selected and the “Run” button was clicked. The optimized purification program was as follows. Briefly, the IMB was mixed for 1 min by moving the magnetic bar sleeve on the mechanical arm up and down. After magnetic separation for 1 min, it was mixed in the incubation well for 5 min by the moving magnetic bar sleeve left and right. Then, the IMB captured with DON was recaptured with a magnetic bar to aggregate to the outer wall of the magnetic bar sleeve from the reaction liquid. The IMB with DON was released and recaptured after suspension in the two wash wells for 1 min. After the IMB with DON was transferred to the eluting well and eluted for 5 min at 100 °C, the IMB was recaptured and released in the second well. Finally, the eluent was accurately removed and filtered through a 0.22 μm PTFE syringe filter before being examined using the UPLC-UV system. 

### 4.8. Immunoaffinity Column Purification

The main operation steps of IAC for DON were summarized as follows, following the manufacturer’s instructions and a reported study [3]. Firstly, 2 mL of the supernatant from “4.6. Sample sources and preparation” was flowed through the IAC at a rate of 2–3 mL/ min to ensure the adequate capture of DON by the IAC. Subsequently, the IAC was, successively, rinsed with 10 mL of PBS, 10 mL of distilled water, and then 2–3 mL of flushing air. Finally, 1 mL methanol followed by 2–3 mL flushing air were used to elute the DON captured by IAC. The collected eluent was dried at 40 °C, under a gentle stream of nitrogen gas, which was then reconstituted with 1 mL acetonitrile/water (10:90, *v*/*v*) and filtered through a 0.22 μm PTFE syringe filter prior to addition to the UPLC-UV system.

### 4.9. Method Validation

The developed method was validated in-house in terms of linearity, limit of detection (LOD), limit of quantification (LOQ), accuracy, precision and selectivity, according to the methods of sampling and analysis in EC Regulation (No. 401/2006) [29]. Linearity was determined with six concentrations of DON between 50 and 1000 ng/mL (50, 100, 250, 500, 750, 1000 ng/mL) and the regression equation including the regression coefficient (R^2^) was calculated by plotting the peak area (*y*) against the concentration of DON (*x*). LOD and LOQ were estimated in triplicate by serially diluted DON standard solution, which produced a chromatogram peak, with 3 and 10 signal-to-noise ratios, respectively. The recovery investigation in triplicate looked into the method’s accuracy and precision, which was conducted by spiking the blank cereals (wheat, maize and husked rice) with various amounts of DON standards (100, 500, 1000, 2000 μg/kg). The result of the recovery study was expressed as recovery rate (%), while the relative standard deviation (RSD, %) was used for precision. Furthermore, the DON reference material from CHARM was used to verify the accuracy of the method. Matrix interference was investigated by comparing chromatograms of DON standard, blank or positive wheat and corn extracts. The selectivity was investigated by 17 common mycotoxins including AFB_1_, AFB_2_, AFG_1_, AFG_2_, AFM_1_, FB_1_, FB_2_, FB_3_, NIV, OTA, ST, T-2 and ZEN, with the aid of UHPLC-HRMS. Matrix interference was investigated by contrast chromatograms of the DON standard, blank or positive wheat and corn extracts.

### 4.10. Data Management

Data were acquired and analyzed using Waters EMPOWER3 software (Waters, Manchester, UK). Statistical analyses during the whole study were performed using Microsoft Excel and the software package OriginPro version 8.5 (OriginLab Corporation, Northampton, USA). The figures were plotted using OriginPro version 8.5. 

## Figures and Tables

**Table 1:** Detected amount and recovery of corn and wheat at different spiking levels (*n* = 3) (RSD: relative standard deviations).

Matrix	Spiked Level (μg/kg)	Detected Amount (μg/kg)	Recovery (%)	RSD (%)
corn	100	98.4 ± 6.9	98.4 ± 6.9	7.0
	500	485.8 ± 28.0	97.2 ± 5.6	5.8
	1000	919.6 ± 51.3	92.0 ± 5.1	5.6
	2000	1840.1 ± 80.8	92.0 ± 4.0	4.4
wheat	100	109.5 ± 4.2	109.5 ± 4.2	3.8
	500	473.1 ± 22.4	94.6 ± 4.5	4.7
	1000	927.2 ± 19.1	92.7 ± 1.9	2.1
	2000	1922.3 ± 120.7	96.1 ± 6.0	6.3

**Table 2:** Detected amount of the deoxynivalenol reference materials (*n* = 3).

DON Reference Material	Lot Number	Detected Amount (μg/kg)	State Value (±, SD, μg/kg)	Target Value (μg/kg)
corn	MRM-DON-CORN-0.5-0021	510.4	520 ± 100	500
	MRM-DON-CORN-1-002G	986.7	1090 ± 20	1000
wheat	MRM-DON-WHEAT-0.5-005A	492.4	510 ± 20	500
	MRM-DON-WHEAT-1-010	943.9	1030 ± 40	1000

## Supplementary Materials

The following are available online at https://www.mdpi.com/article/10.3390/toxins14020093/s1, Table S1. Retention times and accurate masses of selected ions for 18 mycotoxins.
