# Supplementary material for: An Automatic Immunoaffinity Pretreatment of Deoxynivalenol Coupled with UPLC-UV Analysis
Source: Toxins (Basel). 2022 Jan 25;14(2):93. doi: 10.3390/toxins14020093 (PMC8879917; doi:10.3390/toxins14020093)
Supplement: Supplementary file 1 [file toxins-14-00093-s001.zip › toxins-1510566-supplementary.pdf]

Supplementary Materials: An Automatic Immunoaffinity Pretreatment of Deoxynivalenol Coupled with UPLC-UV Analysis

Hongmei Liu, Zhihong Xuan, Jin Ye, Jinnan Chen, Meng Wang, Stephan Freitag, Rudolf Krska, Zehuan Liu, Li Li, Yu Wu and Songxue Wang

Supplementary Table

Table S1. Retention times and accurate masses of selected ions for 17 mycotoxins.

Coumpound	Formula	Retention time (min)	seletcted ion	Expected m/z	Actural m/z	m/z delta (ppm)
NIV	$C_{15}H_{20}O_7$	2.07	$[M+H]^+$	313.12818	313.12796	−0.70193
DON	$C_{15}H_{20}O_6$	4.03	$[M+H]^+$	297.13326	297.13327	0.03454
DON-3G	$C_{21}H_{30}O_{11}$	4.71	$[M+H-Gly]^+$	297.13326	297.13318	−0.27358
3-ADON	$C_{17}H_{22}O_7$	8.69	$[M+H]^+$	339.14383	339.14398	0.44799
15-ADON	$C_{17}H_{22}O_7$	8.93	$[M+H]^+$	339.14383	339.14401	0.53798
AFB_1	$C_{17}H_{12}O_6$	11.57	$[M+H]^+$	313.07066	313.07059	−0.23267
AFB_2	$C_{17}H_{14}O_6$	11.27	$[M+H]^+$	315.08631	315.08627	−0.11681
AFG_1	$C_{17}H_{12}O_7$	11.06	$[M+H]^+$	329.06558	329.06531	−0.82775
AFG_2	$C_{17}H_{15}O_7$	10.51	$[M+H]^+$	331.08123	331.08121	−0.06864
AFM_1	$C_{17}H_{12}O_7$	10.65	$[M+H]^+$	329.06558	329.06543	−0.45679
FB_1	$C_{34}H_{59}NO_{15}$	12.87	$[M+H]^+$	722.39575	722.39569	−0.08179
FB_2	$C_{34}H_{59}NO_{14}$	15.58	$[M+H]^+$	706.40083	706.40082	−0.01717
FB_3	$C_{34}H_{59}NO_{14}$	14.24	$[M+H]^+$	706.40083	706.40057	−0.36278
T-2	$C_{24}H_{34}O_9$	13.82	$[M+NH_4]^+$	484.25411	484.2543	0.39851
ZEN	$C_{18}H_{22}O_5$	14.89	$[M+H]^+$	319.154	319.15381	−0.59973
OTA	$C_{20}H_{18}ClNO_6$	15.37	$[M+H]^+$	404.08954	404.08969	0.37408
ST	$C_{18}H_{12}O_6$	15.54	$[M+H]^+$	325.07066	325.07086	0.62084

Supplementary Methods

Synthesis of Magnetic Beads

Firstly, the magnetic Fe_3O_4 particles were prepared by solvothermal method [20]. 6.0 g of anhydrous sodium acetate and 1.0 g of $FeCl_3{\cdot}6H_2O$ as a ferric source were dissolved in 180 mL glycol under stirring, in order to form a transparent solution. This resultant solution was then transferred into a Teflon-lined stainless-steel autoclave, sealed, and heated at 200 °C for 10 h, and then cooled to room temperature. In order to effectively remove the solvent, the obtained black magnetite particles were collected and washed with ethanol and deionized water (each three times) with the aid of an external magnetic field. The products were dried under vacuum at 60°C for 12 h.

Secondly, 1.0 g freshly prepared Fe_3O_4 particles were redispersed in a mixture of 200 mL ethanol and 100 mL ultrapure water by ultrasonication for 10 min. Then, 1 mL of $NH_3{\cdot}H_2O$ solution (25–28%, w/w) was added under mechanical stirring for 10 min. Afterwards, 4 mL tetraethoxysilane and 20 mL absolute ethyl alcohol were mixed and then added into the above dispersion system at a speed of one drop per second. The reaction proceeded under mechanical stirring overnight at room temperature. The resulting particles

were also collected with a magnet, rinsed with ethanol and ultrapure water three times, and then dried under vacuum at 60°C for 12 h.

Finally, the MBs (Fe_3O_4·SiO_2·Agarose) was prepared by a water/oil emulsion technique using agarose in the aqueous phase and emulsifier (Span-80) as the "oil" phase. 1.0 g Fe_3O_4·SiO_2 particles dispersed in 10 mL water were added in the liquefied agarose solution, which was then added to the above uniform emulsion. After the mixed solution was cooled to 20 °C and mechanically stirred for 1 h, 1 L of ethanol was added to break up the emulsion. Spherical MBs were produced, containing several Fe_3O_4·SiO_2, which was also collected by magnet and washed with ethanol and deionized water five times.

Synthesis of N-Hydroxysuccinimide-Terminated Magnetic Beads

The MB was firstly functionalized with epoxy group by epichlorohydrin activation method. 10 mL of the MAMs was added to 50 mL 1 M NaOH, 30 mL epichlorohydrin, and 50 mL of 1,4-dioxane, and then incubated at 50 ◦C for 12 h with shaking. The obtained particles were washed well with water and ethanol. The epoxy group was introduced onto the agarose of MBs. The product was epoxypropyl functionalized MB with low cross-linking and was also stored in water, with a volume fraction of 50% (*v*/*v*).

Epoxypropyl functionalized MB was further, modified with glycine, which the simplest of the roughly 21 amino acids common in nature. A carboxyl group was introduced onto MB through the reaction between the amino group with the epoxy group. Glycine (2.0 g) and sodium carbonate (2.0 g) were added to 10 mL of 50% (*v*/*v*) epoxypropyl functionalized MB under mechanical stirring overnight at room temperature. After washing well with water and ethanol, the resulting carboxyl activated MB was also stored in water, with a volume fraction of 50% (*v*/*v*).
